# Draft Genome Sequences of Pseudomonas sp. Strains MWU12-2037 and MWU12-2345, Isolated from Peat and Sandy Bog Soils in the Cape Cod National Seashore, Massachusetts

**DOI:** 10.1128/mra.00536-22

**Published:** 2022-07-12

**Authors:** Ayman Anasi, Scott Soby

**Affiliations:** a Biomedical Sciences, College of Graduate Studies, Midwestern University, Glendale, Arizona, USA; b College of Veterinary Medicine, Midwestern University, Glendale, Arizona, USA; University of Arizona

## Abstract

Pseudomonas sp. strains MWU12-2037 and MWU12-2345 were isolated from peat and sandy bog soils in wild cranberry bogs in the Cape Cod National Seashore (Massachusetts, USA) as part of a culture-dependent survey of relatively unexplored wetlands soil microbiomes. Both genomes exceeded 7 Mbp.

## ANNOUNCEMENT

Despite the importance of wetlands bacteria in geochemical processes and ecosystem dynamics, their biological functions are not well understood. The genus Pseudomonas comprises a large number of species that together produce a long list of secondary metabolites that allow adaptation to many different environments (1, 2), but outside agricultural settings, little is known about how these bacteria influence their ecosystems. Pseudomonas sp. strains MWU12-2037 and MWU12-2345 were isolated from peat (42.070624 N, −70.210548 W) and sandy bog (42.064742 N, −70.117562 W) soils in wild cranberry bogs of the Cape Cod National Seashore. Soil cores were vortexed in sterile water, plated onto King’s medium B (KMB) agar plates containing 50 μg mL^−1^ each of cycloheximide and ampicillin, incubated at 26°C for 48 h, colony purified 3×, and stored at −80°C in 34% glycerol. The isolates were recovered from frozen storage on KMB agar; populations were inoculated into overnight KMB broth cultures for genomic DNA (gDNA) isolation using a DNeasy blood and tissue kit (Qiagen). Illumina-compatible gDNA libraries were generated from gDNA with a Kapa Biosystem Hyperplus library kit (KK8514). DNA was enzymatically sheared to ≈500-bp fragments, end-repaired, and A-tailed. Illumina-compatible adapters with unique indexes (IDT 00989130v2) were individually ligated to each sample, cleaned using Kapa Biosciences pure beads (KK8002), and amplified with Kapa HiFi enzyme (KK2502). The libraries were analyzed for fragment size using the TapeStation system (Agilent Technologies), quantified by quantitative PCR (qPCR) using a Kapa library quantification kit (KK4835; Thermo Fisher Scientific; QuantStudio 5), and then multiplex-pooled and sequenced using an Illumina MiSeq device on a 2 × 250-bp flow cell. The raw reads were assembled using Unicycler v0.4.8 (3) and polished using Pilon v1.23 (4) within the PATRIC (http://patricbrc.org) Comprehensive Genome Analysis v3.6.12 pipeline using default settings, except for the trim function, which was set to “true” for quality control and trimming using Trim Galore v0.4.0 (https://www.bioinformatics.babraham.ac.uk/projects/trim_galore/) (5, 6). The genome sequences were annotated using RASTtk (7) as part of the PATRIC pipeline. Using the Type (Strain) Genome Server (https://tygs.dsmz.de/), isolates were placed within the genus Pseudomonas with high confidence using the Genome BLAST Distance Phylogeny approach (8) and were found to be closely related (digital DNA-DNA hybridization [dDDH_d4_] score = 86.6%).

MWU12-2037 had an assembled genome size of 7,222,596 bp distributed over 57 contigs, 61.34% G+C content from 1,956,500 reads, and a read length of 909,223,076 bp. The sequence coverage was 125×, with an *N*_50_ value of 422,677 bp. There were 4,934 protein-coding, 60 tRNA, and 4 rRNA genes. MWU12-2345 had an assembled genome size of 7,382,352 bp distributed over 57 contigs, 61.22% G+C content from 1,552,993 reads, and a read length of 712,111,537 bp. The sequence coverage was 96×, with an *N*_50_ value of 335,205 bp. There were 5,063 protein-coding, 61 tRNA, and 5 rRNA genes.

### Data availability.

This whole-genome shotgun project for MWU12-2037 and MWU12-2345 has been deposited at DDBJ/ENA/GenBank under accession numbers JALLIZ000000000 and JALLJA000000000 and BioSample accession numbers SAMN26856950 and SAMN26856949, respectively. The versions described are JALLIZ000000000.1 and JALLJA000000000.1, respectively. The BioProject accession number is PRJNA691338. The SRA accession numbers are SRR18508470 for MWU12-2037 and SRR18508469 for MWU12-2345. The RASTtk annotations are available under open license at Zenodo (https://zenodo.org/record/6441768#.Yl9mFtrMLD4 and https://zenodo.org/record/6441878#.Yl9mFNrMLD4).
